# High‐sensitivity C‐reactive Protein is a Predictive Factor of Adiposity in Children: Results of the Identification and prevention of Dietary‐ and lifestyle‐induced health Effects in Children and InfantS (IDEFICS) Study

**DOI:** 10.1161/JAHA.113.000101

**Published:** 2013-06-21

**Authors:** Annunziata Nappo, Licia Iacoviello, Arno Fraterman, Esther M. Gonzalez‐Gil, Charis Hadjigeorgiou, Staffan Marild, Denes Molnar, Luis A. Moreno, Jenny Peplies, Isabel Sioen, Toomas Veidebaum, Alfonso Siani, Paola Russo

**Affiliations:** 1 Unit of Epidemiology and Population Genetics, Institute of Food Sciences, National Research Council, Avellino, Italy (A.N., A.S., P.R.); 2 Laboratory of Genetic and Environmental Epidemiology, Research Laboratories, Fondazione di Ricerca e Cura “Giovanni Paolo II”, Università Cattolica, Campobasso, Italy (L.I.); 3 Medizinisches Versorgungszentrum Dr Eberhard &Partner Dortmund, Laboratoriumsmedizin Dortmund, Dortmund, Germany (A.F.); 4 Growth, Exercise, Nutrition and Development (GENUD) Research Group, University of Zaragoza, Zaragoza, Spain (E.M.G.G., L.A.M.); 5 Research and Education Institute of Child Health, Strovolos, Cyprus (C.H.); 6 Department of Pediatrics, Queen Silvia Children's Hospital, University of Gothenburg, Gothenburg, Sweden (S.M.); 7 Department of Pediatrics, Medical Faculty, University of Pecs, Pécs, Hungary (D.M.); 8 BIPS – Institute for Epidemiology and Prevention Research, Bremen, Germany (J.P.); 9 Department of Public Health, Faculty of Medicine and Health Sciences, Ghent University, Ghent, Belgium (I.S.); 10 Research Foundation – Flanders, Brussels, Belgium (I.S.); 11 National Institute for Health Development, Tallinn, Estonia (T.V.)

**Keywords:** C‐reactive protein, inflammation, pediatric obesity, population

## Abstract

**Background:**

Whereas cross‐sectional studies have shown that obesity is associated with increased C‐reactive protein (CRP) levels in children, little is known about the impact of low‐grade inflammation on body mass changes during growth.

**Methods and Results:**

We assessed cross‐sectionally and longitudinally the association of high‐sensitivity (hs)‐CRP levels with overweight/obesity and related cardiometabolic risk factors in the Identification and prevention of Dietary‐ and lifestyle‐induced health Effects in Children and InfantS (IDEFICS) cohort. 16 224 children from 8 European countries (2 to 9 years) were recruited during the baseline survey (T0). After the exclusion of 7187 children because of missing hs‐CRP measurements and 2421 because of drug use during the previous week, the analysis was performed on 6616 children (Boys=3347; Girls=3269; age=6.3±1.7 years). Of them, 4110 were reexamined 2 years later (T1). Anthropometric variables, blood pressure, hs‐CRP, blood lipids, glucose and insulin were measured. The population at T0 was divided into 3 categories, according to the baseline hs‐CRP levels. Higher hs‐CRP levels were associated with significantly higher prevalence of overweight/obesity, body mass index (BMI) z‐score and central adiposity indices (*P* values all <0.0001), and with higher blood pressure and lower HDL‐cholesterol levels. Over the 2‐year follow‐up, higher baseline hs‐CRP levels were associated with a significant increase in BMI z‐score (*P*<0.001) and significantly higher risk of incident overweight/obesity.

**Conclusions:**

Higher hs‐CRP levels are associated to higher body mass and overweight/obesity risk in a large population of European children. Children with higher baseline levels of hs‐CRP had a greater increase in BMI z‐score and central adiposity over time and were at higher risk of developing overweight/obesity during growth.

## Introduction

The role of chronic low‐grade inflammation as a possible link between obesity and its metabolic and cardiovascular sequelae has been convincingly showed during the last years.^1^

C‐reactive protein (CRP) has long been considered a prototypic biomarker of subclinical inflammation.^2^ Since analytical standards were defined for its measurement,^3^ the high‐sensitivity CRP (hs‐CRP) assay is considered a sensitive predictor of atherosclerosis, diabetes and coronary heart diseases also in apparently healthy individuals.^4–7^

About 10 years ago, large epidemiological studies unequivocally showed the association of low‐grade inflammation and overweight in children.^8–10^ The number of studies exploring different aspects of this association has been steadily increasing in the last years, as recently reviewed by Tam et al.^11^

The summary evidence from previous studies is that a consistent association exists between obesity and increased CRP levels in children. However, since most of the information derives from cross‐sectional investigations, there is a need to confirm this association in longitudinal studies, aiming to confirm the value of hs‐CRP as an early‐detectable surrogate marker in the natural history of the association between childhood obesity and cardiovascular disease late in adulthood.

The aim of the present study was to assess cross‐sectionally and longitudinally the association of hs‐CRP levels with overweight/obesity and related cardiometabolic risk factors in a large sample of European preschool and primary school children participating in the IDEFICS project. The 2‐year follow‐up of the Identification and prevention of Dietary‐ and lifestyle‐induced health Effects in Children and InfantS (IDEFICS) cohort allowed to test whether higher baseline levels of hs‐CRP were associated with higher body mass and increased incidence of overweight/obesity over time.

## Materials and Methods

### Ethics Statement

The study was conducted according to the standards of the Declaration of Helsinki. All applicable institutional and governmental regulations pertaining to the ethical use of human volunteers were followed during this research. Approval by the appropriate ethics committees was obtained by each of the 8 participating centers carrying out the fieldwork. Participants were not subjected to any study procedure before both the children and their parents gave their oral (children) and written (parents) informed consent for examinations, collection of samples, subsequent analysis and storage of personal data and collected samples.

### Study Population

IDEFICS is a large European multicenter study of childhood obesity. Details of the general design, instruments and survey characteristics can be found elsewhere.^12^

A cohort of 16 224 children aged 2 to 9 years was recruited into a population‐based baseline survey from 8 European countries ranging from the north to the south and from the east to the west (Belgium, Cyprus, Estonia, Germany, Hungary, Italy, Spain, and Sweden). The baseline survey (T0) was the starting point of the cohort study aimed to prospectively evaluate the role of the factors assessed at baseline on the development of overweight/obesity over time, and to assess the feasibility, effectiveness, and sustainability of a community‐oriented intervention program. Comparable intervention and control regions were selected in each country. In the intervention regions, a coherent set of intervention modules were implemented, focusing on diet, physical activity, and stress‐coping capacity. All the materials for the interventions were centrally developed and culturally adapted. A detailed description of the IDEFICS intervention program has been recently published.^13^

A second survey (T1) reassessed the children 2 years later. The follow‐up survey was synchronized with the baseline to account as much as possible for seasonal variation. In fact, the study protocol, closely followed by each survey center, allowed for a 2‐year period (±1 month) between T0 and T1.

Of the 16 224 children examined at T0, 7187 children were excluded from the present analysis. In particular, 4766 children had no hs‐CRP measured (Boys=50%; age=5.8± 1.8 years; mean±SD) and 2421 children (Boys=53%; age=5.9±1.8 years) were excluded because of the use of medications during the week previous to the visit. Eventually, the cross‐sectional analysis was carried out in 6616 children (Boys=50%; age=6.3±1.7 years). Excluded children were significantly younger than those included (*P*<0.001), likely due to the lower acceptance of blood drawing in younger children, while anthropometric parameters were not statistically significant different between the 2 groups (data not shown).

Of these 6616 children, 4110 were re‐examined 2 years later (T1) (Boys=51%; age=8.2±1.7 years, control group n=1960, intervention n=2150). The distribution of children in the control and intervention region was balanced across the baseline hs‐CRP categories. Since no statistically significant differences in the anthropometric outcomes were observed between children in the intervention and in the control regions, longitudinal analyses were performed on the whole T1 population sample.

### Anthropometry and Blood Pressure

Children underwent a standardized physical examination. Anthropometric data included body weight and height, waist and hip circumferences, and the measurement of skinfold thickness. A detailed description of the anthropometric measurements in the IDEFICS study, including intra‐ and interobserver reliability, has been recently published.^14^

The measurement of weight was carried out using an electronic scale (Tanita BC 420 SMA, Tanita Europe GmbH) to the nearest 0.1 kg with children wearing light clothes without shoes. Height was measured using a telescopic height‐measuring instrument (Seca 225 stadiometer) to the nearest 0.1 cm. Body mass index (BMI) was calculated as weight (in kg) divided by height squared (in m). For each child, z‐scores of BMI and of BMI variation over the 2‐year follow‐up were determined according to the sex‐ and age‐specific z‐scores by Cole et al.^15^

Waist circumference was measured using an inelastic tape (Seca 200), precision 0.1 cm, range 0±150 cm at the midpoint between the iliac crest and the lower border or tenth rib with the subject in a standing position and recorded at the nearest 0.1 cm. Both triceps and subscapular skinfold thickness were measured by means of a caliper (Holtain, Holtain Ltd, range 0±40 mm). Measures were taken twice on the right hand side of the body and the mean was calculated.

For the definition of overweight/obesity, children were grouped into 2 categories using the cut‐points defined by Cole et al^16–17^: (1) underweight (thinness grade III to I) plus normal weight and (2) overweight plus obese.

Systolic and diastolic blood pressure (BP) was measured after 5 minutes rest in the seated position, at the child right arm with a cuff of appropriate size for arm circumference using an automatic sphygmomanometer (Welch Allyn 4200B‐E2 Inc.). Two measurements were made and the average of the measurements was used for analysis.

### Biochemistry

Children were asked to participate, on voluntary basis, in blood drawing. A detailed description of sample collection and analytical procedures in the IDEFICS survey has been recently published.^18–19^

CRP levels were measured in a central laboratory with a high‐sensitivity assay using latex‐enhanced nephelometry (BN2‐Nephelometer, Siemens), The lower limit of detection of the assay was 0.02 mg/dL.

Blood glucose, total‐ and HDL‐cholesterol, and triglycerides were assessed, in fasting status by the portable Cholestech LDX analyzer at each point‐of‐care center.^20^ LDL‐cholesterol was calculated according to the formula of Friedwald.^21^

Serum insulin and glycated hemoglobin (HbA1c) were measured in a central laboratory through enzyme‐linked immunosorbent assay kit (MODULAR E170, Roche Diagnostics) and by high‐pressure liquid chromatography with an automated HLC‐723G7 analyzer respectively. Homeostatic model assessment (HOMA) index was calculated according to the formula: HOMA = [serum insulin (μU/mL) × blood glucose (mmol/L)]/22.5.^22^ Categories of “insulin‐resistant” children were defined by the 75th percentile of HOMA index for boys (normal, HOMA index≤1.16, high >1.16) and girls (normal, HOMA index≤1.24, high >1.24).

### Questionnaire

Parents were asked to fill in a questionnaire to collect data on the participating child (date of birth, weight and length at birth, physical activity and lifestyle factors, personal and familial medical history). Parental information was collected as well (age, self‐reported weight and height, education level, and occupation). With regard to physical activity, parents were invited to indicate how many minutes per day their child used to spend in outdoor activities (ie, time spent playing in the yard or street around house or outdoor recreation area [swimming pool, zoo, park] either on week days or weekend days). An additional question investigated whether or not the child would have attended a club to practice sport. The total weekly physical activity time was calculated as the sum of the number of hours spent in each activity category. Educational level of the parents was estimated by International Standard Classification of Education (ISCED) criteria ^23^ in 6 categories (from 0 to 5). Parents were considered overweight if their self‐reported BMI was ≥25 kg/m^2^. Breastfeeding was classified as: none; 0 to 3 months; 3 to 6 months; >6 months. Parents were also asked to indicate whether their child had taken any kind of medication, including self‐prescribed drugs, vitamin, and mineral supplements, during the week before blood sample drawing.

### Statistical Analysis

Statistical analyses were performed using PASW (Predictive Analytics SoftWare) Statistics (version 18; SPSS Inc.). The analyses were performed separately in boys and girls. Data are presented as mean (95% confidence intervals) or median and range, as indicated. Categorical variables are shown as absolute frequencies. Given that the distribution of hs‐CRP was skewed, log‐transformed values were used for statistical analysis. For ease of interpretation, untransformed values are presented. Because about 50% of children had values less than the minimal detectable concentration, in this analysis hs‐CRP is treated as a categorical rather than as a continuous variable. The following 3 hs‐CRP cut–offs were arbitrarily defined to categorize hs‐CRP levels in our population: (I) hs‐CRP under the detection limit (<0.02 mg/dL); (II) hs‐CRP>0.02 mg/dL and <75th sex‐specific percentile of those with hs‐CRP values over the detection limit (<0.177 mg/dL in boys and 0.180 mg/dL in girls) (III) hs‐CRP>75th sex‐specific percentile of those with hs‐CRP values over the detection limit (>0.177 mg/dL in boys and >0.180 mg/dL in girls). Prevalence of overweight/obesity across hs‐CRP categories was tested by linear by linear association chi‐square test statistics.

Multiple linear regression analysis (GLM) was used to compare mean values of outcome variables across categories of hs‐CRP. *P* values for linear trend were calculated. All analyses were adjusted for child age, country of origin, breast‐feeding, physical activity, parental overweight/obesity (none or at least one affected parent), and parental education. BP and metabolic variables were also adjusted for BMI.

Longitudinal analyses were performed using the 2‐year variation in the outcome adiposity variables (follow‐up, T1 minus baseline, T0) across the baseline hs‐CRP categories, by multiple linear regression analysis adjusted for the respective baseline value of the outcome variable and for other relevant confounders (age, country of origin, physical activity, parental overweight and obesity, breast‐feeding, parental education, intervention/control study group). P values for linear trend were calculated. Incident cases of overweight/obesity over the 2‐year follow‐up according to hs‐CRP groups at baseline were assessed by logistic regression analysis, taking into account age, country of origin, and intervention/control study group. Odds ratio and their 95% confidence intervals were calculated. A 2‐tailed *P* value less than 0.05 was considered statistically significant.

## Results

### Cross‐sectional Analysis

Baseline anthropometric and metabolic characteristics of the male and female participants are summarized in Table 1. Hs‐CRP levels ranged between 0.02 mg/dL (detection limit) and 8.50 mg/dL in boys and 9.35 mg/dL in girls, respectively.

**Table 1.** Characteristics of the Study Subjects by Sex

	Boys (n=3347)	Girls (n=3269)
Age, y	6.22±1.76	6.31±1.73
BMI, kg/m^2^	16.45±2.50	16.45±2.49
BMI z‐score	0.26±1.31	0.24±1.38
Sum of skinfolds, mm	17.17±7.58	19.95±8.11
Waist circumference, cm	54.92±7.06	54.43±6.93
SBP, mm Hg	100.6±9.2	100.4±9.2
DBP, mm Hg	62.6±6.6	63.4±6.5
Glucose, mmol/L	4.72±0.54	4.62±0.52
HbA1c, %	4.67±0.56	4.61±0.58
Insulin, pmol/L	28.89±24.64 (n=2905)	33.20±27.39 (n=2777)
HOMA‐Index	0.90±0.83 (n=2732)	1.00±0.87 (n=2592)
TG, mmol/L	0.48±0.27	0.50±0.28
Total Cholesterol, mmol/L	4.10±0.76	4.22±0.82
HDL‐Cholesterol, mmol/L	1.39±0.37	1.35±0.37
hs‐CRP (mg/dL), median (range)	0.03 (0.02 to 8.50)	0.04 (0.02 to 9.35)

Values are mean±standard deviation or median and range. BMI indicates body mass index; SBP, systolic blood pressure; DBP, diastolic blood pressure; HbA1c, glycosylated hemoglobin A 1c; HOMA, Homeostatic model assessment; TG, triglycerides; HDL, high‐density lipoprotein, hs‐CRP, high sensitivity C‐reactive protein.

Table 2 shows the anthropometric and metabolic variables at baseline (T0) according to the 3 categories of hs‐CRP in boys and girls. The prevalence of overweight/obesity significantly increased across the categories of hs‐CRP in both sexes (Table 2). At multiple regression analysis, adjusted for age, country of origin, breast‐feeding, physical activity, parental overweight/obesity, and parental education, a linear highly significant increase in BMI z‐score, waist circumference, and sum of skinfolds was observed both in boys and in girls, moving across the categories of hs‐CRP.

**Table 2.** Anthropometric and Biochemical Variables in Boys and Girls Across the 3 Categories of hs‐CRP at Baseline (T0)

	Boys				Girls			
	I	II	III	*P* Value for Trend	I	II	III	*P* Value for Trend
	<0.020 mg/dL	>0.020<0.177 mg/dL	>0.177 mg/dL		<0.020 mg/dL	>0.020<0.180 mg/dL	>0.180 mg/dL	
N	1535	1359	453		1095	1608	566	
Age, y	6.38±0.04	6.15±0.05	5.91±0.08	<0.0001	6.44±0.05	6.24±0.04	6.24±0.07	0.008
OW/OB, %	8.3	23.3	32.2	χ²≤0.0001*	7.0	24.9	38.0	χ²≤0.0001*
BMI z‐score	−0.059±0.033	0.430±0.035	0.611±0.062	<0.0001	−0.288±0.041	0.366±0.034	0.688±0.057	<0.0001
WC, cm	53.3±0.2	55.6±0.2	57.5±0.3	<0.0001	52.1±0.2	54.8±0.2	57.2±0.2	<0.0001
SS, mm	15.03±0.19	18.02±0.20	21.33±0.35	<0.0001	16.74±0.23	20.42±0.19	24.11±0.33	<0.0001
SBP, mm Hg*	100.1±0.2	101.1±0.2	100.7±0.4	0.03	100.3±0.3	100.2±0.2	100.8±0.4	NS
DBP, mm Hg*	62.3±0.2	62.9±0.2	63.2±0.3	0.004	63.0±0.21	63.5±0.17	64.0±0.30	0.007
TG, mmol/L*	0.48±0.01	0.48±0.01	0.48±0.01	NS	0.48±0.09	0.50±0.07	0.49±0.01	NS
TC, mmol/L*	4.17±0.02	4.10±0.02	3.87±0.04	<0.0001	4.31±0.03	4.26±0.02	3.98±0.04	<0.0001
HDL‐C, mmol/L*	1.43±0.01	1.38±0.01	1.32±0.02	<0.0001	1.40±0.01	1.37±0.01	1.25±0.02	<0.0001
TC/HDL‐C	3.10±0.03	3.17±0.03	3.16±0.06	NS	3.27±0.04	3.32±0.03	3.49±0.06	0.005
Glucose, mmol/L*	4.74±0.01	4.71±0.02	4.63±0.03	0.001	4.62±0.02	4.59±0.01	4.61±0.02	NS
HbA1c, %*	4.65±0.02	4.70±0.02	4.69±0.03	NS	4.59±0.02	4.62±0.02	4.58±0.03	NS
HOMA Index*	0.92±0.02	0.87±0.02	0.85±0.04	NS	1.02±0.03	0.95±0.02	0.99±0.04	NS
Insulin, pmol/L*	28.54±0.65	27.99±0.69	27.85±1.24	NS	33.40±0.85	31.53±0.71	33.54±1.22	NS

Value are mean±standard error. Multiple regression trend analysis for age, country of origin, physical activity, parental overweight and obesity, breast‐feeding, and parental education. OW/OB indicates overweight/obesity; BMI, body mass index; WC, waist circumference; SS, sum of skinfolds; SBP, systolic blood pressure; DBP, diastolic blood pressure; TG, triglycerides; TC, total cholesterol; HDL‐C, high‐density lipoprotein cholesterol; HbA1c, glycosylated hemoglobin A 1c; HOMA, Homeostatic model assessment; NS, not significant.

* Adjusted for the same variables as above plus BMI.

* *P* by Linear‐by‐linear association.

After adjustment for BMI, a significant increase in diastolic blood pressure was observed in both sexes, while higher systolic blood pressure values in the higher hs‐CRP categories were observed only in boys. In both sexes higher values of hs‐CRP were associated with lower total cholesterol and HDL‐cholesterol levels. The ratio between total and HDL‐cholesterol was significantly higher in girls with higher hs‐CRP levels. Indices of glucose status/insulin‐resistance did not vary across hs‐CRP categories in both sexes, but for the lower plasma glucose levels observed in the boys with higher hs‐CRP values. At categorical analysis, the proportion of children with higher insulin resistance (HOMA index > 75th percentile of the distribution for boys and girls respectively) did not differ across the categories of hs‐CRP.

### Prospective Analysis

Among the 6616 children examined at T0, 4110 were reexamined 2 years later (T1) (62% of the T0 sample). Table 3 shows the prevalence of overweight/obesity and the adjusted changes in anthropometric variables over the 2‐year follow‐up across the hs‐CRP categories assessed at baseline (T0). In both boys and girls, over the 2‐year follow‐up, participants with higher baseline hs‐CRP levels showed significantly higher increase in BMI z‐score and indices of central adiposity, after adjustment for the respective baseline value and independently of the study group (control versus intervention). The highly significant statistical association between baseline hs‐CRP values and the variation of BMI over the 2‐year follow‐up was indeed not affected by the intervention (*P* for trend: boys, unadjusted=0.000066; adjusted=0.000062; girls, unadjusted=0.001; adjusted=0.001).

**Table 3.** Prevalence of Overweight/Obesity and Changes in Anthropometric Variables Over the 2‐Year Follow‐up Across the hs‐CRP Categories

		Boys				Girls			
		I	II	III	*P* Value for Trend	I	II	III	*P* Value for Trend
		<0.020 mg/dL	>0.020<0.177 mg/dL	>0.177 mg/dL		<0.020 mg/dL	>0.020<0.180 mg/dL	>0.180 mg/dL	
N		963	856	278		694	992	327	
OW/OB, %		12.1	28.2	36.3	χ²≤0.0001*	8.5	25.6	37.3	χ²≤0.0001*
Δ BMI z‐score		0.107±0.022	0.196±0.023	0.279±0.041	<0.0001	0.071±0.027	0.188±0.022	0.235±0.039	<0.001
Δ SS, mm		2.046±0.166	3.018±0.172	3.058±0.312	0.0001	2.682±0.194	3.260±0.159	3.404±0.281	0.019
Δ WC, cm		4.150±0.122	4.614±0.127	4.658±0.227	0.011	4.013±0.154	4.424±0.125	5.006±0.220	0.0003

Values are mean±standard error. Multiple regression analysis adjusted for the respective baseline value of the outcome variable and for other relevant confounder (age, country of origin, physical activity, parental overweight and obesity, breast‐feeding, parental educational, intervention/control study group). OW/OB indicates overweight/obesity; BMI, body mass index; SS, sum of skinfolds; WC waist circumference.

* *P* by Linear‐by‐linear association.

Out of 4110 children reexamined at T1, 1951 boys and 1819 girls were not overweight/obese at baseline (T0). After the 2‐year follow‐up, 179 incident cases of overweight/obesity were observed in boys and 137 in girls. At logistic regression analysis−adjusted for age, country of origin, physical activity, parental overweight and obesity, breast‐feeding, parental education, and intervention/control study group−the risk to become overweight/obese, according to the baseline hs‐CRP levels, increased significantly across the hs‐CRP categories (Table 4).

**Table 4.** Odds Ratio to Develop Overweight/Obesity Over the 2‐year Follow‐Up by hs‐CRP Categories Defined at Baseline (T0)

	Boys (n=1952)			Girls (n=1819)		
	OR	(95% CI)	*P* Value	OR	(95% CI)	*P* Value
I	1	—	—	1	—	—
II	2.0	(1.4;2.9)	<0.001	2.0	(1.2;3.2)	0.004
III	2.0	(1.1;3.5)	0.022	2.6	(1.4;4.7)	0.002

Overweight/obesity defined according to International Obesity Task Force age‐ and sex‐specific cut‐off. Logistic regression analysis adjusted for age, country of origin, physical activity, parental overweight and obesity, breast‐feeding, parental education, and intervention/control study group. OR indicates odds ratio; CI, confidence interval. hs‐CRP value: Boys I: <0.020 mg/dL; II: >0.020<0.177 mg/dL; III: >0.177 mg/dL— Girls I: <0.020 mg/dL; II: >0.020<0.180 mg/dL; III: >0.180 mg/dL.

## Discussion

The current study evaluated the association between hs‐CRP levels, adiposity indices and cardiometabolic abnormalities in the preschool and primary school children participating in the European IDEFICS cohort. The longitudinal evaluation allowed the current study to focus on the predictive value of baseline hs‐CRP levels on the changes in anthropometric parameters and on the development of overweight/obesity over the 2‐year follow‐up. Cross‐sectional analyses revealed that boys and girls with higher plasma concentrations of hs‐CRP at baseline present (1) higher BMI z‐scores and central fat deposition; (2) higher prevalence of overweight/obesity; (3) higher BMI‐adjusted BP and lower BMI‐adjusted HDL‐cholesterol levels, with a significantly worse ratio between total and HDL‐cholesterol in girls. Longitudinal analyses showed that higher plasma concentrations of hs‐CRP at baseline predict (1) a higher increase of BMI z‐scores and central adiposity indexes over the 2‐year follow‐up, and (2) a significantly higher risk of incident overweight/obesity in both boys and girls.

Our data showed a strong correlation between adiposity indices and hs‐CRP in children. This correlation was similar to other studies where plasma hs‐CRP levels were positively associated with adiposity in adults ^24–26^ and children.^8–10,27^ In our study, cross‐sectional analyses also showed that higher hs‐CRP values are associated with cardiovascular risk factors, like higher BP and lower HDL‐cholesterol levels. Previous studies already suggested that high CRP levels in children are associated with cardiovascular risk factors including HDL‐cholesterol and BP,^8,28–29^ although in some studies the association did not survive after adjustment for body mass.^29–30^ In our study, the low‐grade proinflammatory status consistent with higher hs‐CRP levels is associated to a worse atherogenic profile already in preschool and school children, independently from body mass. The finding is of interest because the low‐grade inflammation has been found to predict the development of CVD ^31^ and type 2 diabetes.^6^

With regard to insulin resistance, Shea et al^32^ and Moran et al^33^ provided evidence of an association of CRP levels with insulin resistance markers in children, although no longer significant after adjustment for adiposity in the study by Moran.^33^ In our study we did not observe differences in insulin resistance measures between the different groups of hs‐CRP, probably due to the fact that our cohort is on average too young for detecting some metabolic derangements that might occur more likely later with age.^34–35^ In fact, the International Diabetes Federation and the American Heart Association have both proposed a definition of metabolic syndrome in children and adolescents.^34–35^ In both guidelines, the cut‐offs for metabolic and BP variables were defined only for children above 10 years of age, due to the high variability of these variables below this age. It is thus possible that the association would be more evident during adolescence rather than childhood. Long‐term prospective studies, as the ongoing follow‐up of the IDEFICS cohort, are needed to answer this question.

A strength of the present study is the use of precisely standardized phenotypic measurements within the 8 European countries participating in the survey. In fact, all measurements were conducted according to detailed standard operation procedures. In particular, subsamples of study subjects were examined repeatedly to calculate the inter‐ and intraobserver reliability of anthropometric measurements.^14^

The primary limitation is the use of a single hs‐CRP measurement. In adults, repeat hs‐CRP measurements are recommended, as concentrations are affected by factors such as recent infection and inflammatory conditions. The measurement of other inflammatory biomarkers could have provided further insights on the early association between inflammation and body fat accumulation. Other tests for active infection, for example cytokines or white blood cell count, were not performed, although the exclusion from the analysis of children who took medications during the week before the blood drawing may be considered as an adequate proxy. The assessment of the parental smoking and the consequent secondary smoking exposure of children would have been of interest, but unfortunately these questions were not included in the IDEFICS questionnaire. A limitation of the longitudinal analysis is the lower number of children attending the follow‐up examination and the relatively short duration of the follow‐up. However, the longitudinal association of basal hs‐CRP values with adiposity indices and incidence of overweight/obesity clearly emerged even after the relatively short follow‐up period and notwithstanding the expected reduction of the sample size, mainly due to the burden of the extensive examination protocol. The comparability of the core measurements between the children retained and those lost at follow‐up makes us confident that selection bias did not affect the longitudinal results. The planned follow‐up of the IDEFICS cohort in subsequent years will allow further evaluation of the association of hs‐CRP with body mass and metabolic variables during the transition from childhood to adolescence. Finally, the possibility that the intervention program conducted in half of the population could have marginally affected the relationship between the baseline hs‐CRP values and the phenotypes of interest over the follow‐up cannot be excluded. This possibility was taken into account by adjusting for the control/intervention group in the longitudinal analysis. In summary, we confirmed the association between hs‐CRP and higher body mass and overweight/obesity risk in a large population of European children. We also showed that hs‐CRP levels are associated to a worse cardiovascular risk profile. The main and novel finding of the study is that the children with higher baseline level of hs‐CRP had a greater increase in body mass and central adiposity over time and therefore were at higher risk of developing overweight/obesity during growth, as compared with children less exposed to low‐grade inflammation.
